# Exploiting Herpes Simplex Virus Entry for Novel Therapeutics

**DOI:** 10.3390/v5061447

**Published:** 2013-06-10

**Authors:** Satvik Hadigal, Deepak Shukla

**Affiliations:** 1 Department of Ophthalmology and Visual Sciences, College of Medicine, University of Illinois at Chicago, 1855 West Taylor Street, m/c 648, Room 3.138, Chicago, IL 60612, USA; E-Mail: satvikhadigal@gmail.com; 2 Department of Microbiology and Immunology, College of Medicine, University of Illinois at Chicago, 835 S. Wolcott, Chicago, IL 60612, USA; 3 Lions of Illinois Eye Research Institute, University of Illinois at Chicago, 1905 West Taylor Street, Chicago, IL 606012, USA

**Keywords:** herpes, HSV-1, HSV-2, therapeutics, peptides, entry, G1 and G2, aptamers, dendrimers, AC-8, dermaseptins, PI3K inhibitor, K-5 compounds, nanoparticles, compound SP-510-50, apolipoprotein E, viral glycoproteins, gB, gD, gH/gL, viral surfing, hemifusion, fusion, endocytosis

## Abstract

Herpes Simplex virus (HSV) is associated with a variety of diseases such as genital herpes and numerous ocular diseases. At the global level, high prevalence of individuals who are seropositive for HSV, combined with its inconspicuous infection, remains a cause for major concern. At the molecular level, HSV entry into a host cell involves multiple steps, primarily the interaction of viral glycoproteins with various cell surface receptors, many of which have alternate substitutes. The molecular complexity of the virus to enter a cell is also enhanced by the existence of different modes of viral entry. The availability of many entry receptors, along with a variety of entry mechanisms, has resulted in a virus that is capable of infecting virtually all cell types. While HSV uses a wide repertoire of viral and host factors in establishing infection, current therapeutics aimed against the virus are not as diversified. In this particular review, we will focus on the initial entry of the virus into the cell, while highlighting potential novel therapeutics that can control this process. Virus entry is a decisive step and effective therapeutics can translate to less virus replication, reduced cell death, and detrimental symptoms.

## 1. Introduction

Herpes simplex virus (HSV) is a double-stranded DNA virus, belonging to Alphaherpesvirinae family, a subfamily of the Herpesviridae family. There are two highly related serotypes of HSV: HSV‑1 and HSV-2. The former is a significant cause of ocular disease, and 70%–90% of the population is seropositive for this virus [1]. Ocular HSV-1 infection may manifest in different clinical situations, including as conjunctivitis, iridocyclitis, acute retinal necrosis, or keratitis. There has also been a recent surge of evidence suggesting of HSV-1 involvement in Alzheimer’s disease (AD). As AD pathogenesis now has a possible link to the reactivation of latent HSV-1 virus in the trigeminal ganglion [2]. HSV-2, on the other hand, predominantly causes genital herpes, although some HSV-1 strains have also been reported to cause genital infection [3]. 

HSV infections of both types are common in both the industrialized and developing worlds. The virus has the ability to successfully avoid the host immune system by entering a non-replicating state known as latency. This establishes a lifelong infection with periods of reactivation that are stimulated by various environmental cues [4,5]. Prevention of infection is particularly difficult, as transmission between individuals often occurs during unrecognized and asymptomatic shedding during latency in the host individual. 

The success of HSV, particularly noted in the high percentage of the population that is seropositive for either HSV-1 or HSV-2 [1], is aided by its ability to infect multiple cell types via exploiting various receptors. The HSV viral envelope is composed of about a dozen viral glycoproteins in a lipid bilayer, many of which have the ability to use multiple alternative host cell-receptors. In addition, the virus has multiple strategies to enter the cell, including using fusion or different forms of endocytosis. HSV entry is a crucial stage in infection and cell-to-cell spread, in the lifecycle of the virus. It is difficult to eliminate the virus after this initial stage of infection with current therapeutics [6]. For example, nucleoside analogs acyclovir and similarly functional triflutothymidine (TFT) require phosphorylation; therefore their antiviral activity is not initiated until the infection has progressed to the point that viral thymidine kinase has been synthesized. Thus, new treatments targeting prevention of entry will be more useful in stopping the transmission of the virus to new hosts. 

This review will focus on the primary interaction and entry of the virus into host cells. An improved understanding of the viral processes could have a signiﬁcant effect on prevention and treatment. In addition, several potential prophylactics that target this stage of infection are also discussed. 

## 2. Entry

### 2.1. Attachment and Surfing

The initial step in HSV entry is attachment to the plasma membrane of host cell. The purpose of this step is to concentrate the virus on the cell surface, not to necessarily trigger fusion. The primary attachment receptors for HSV are glycoprotein B (gB) and glycoprotein C (gC), both of which bind to heparan sulfate proteoglycans (HSPGs). Glycoproteins B and C of HSV-1 and HSV-2 have different affinities to HSPGs. gC has a critical role in HSPG attachment during HSV-1 entry [7], while gB is the crucial glycoprotein for HSV-2 in this step [8]. Furthermore, gB and gC have both been found to bind to different structures on heparan sulfate (HS) [9].

Binding to cells, and thus infectivity, is severely reduced in gB and gC deficient viruses [10]. gC deficient viruses are capable of infecting cells, although to a lesser degree [11]. gC merely enhances HSV binding through its interaction with heparan sulfate, but is not essential for entry [12]. gB plays a further significant role in entry as it is responsible for fusion pore formation in a later step.

After initial attachment to HS, the virus, when not in the vicinity of fusion receptors, travels to the cell body via lateral movement along the length of filopodia, in a process described as “surfing”. Filopodia are thin, rod-like cell surface extensions formed by bundles of parallel actin filaments [13]. Due to the greater surface area of protrusions, transient viruses have a higher chance of attaching to filopodia than to the cell surface. The lack of fusion receptors on filopodia engages viruses to “surf” and gain access to gD receptors present on the cell body. In addition to providing a preliminary docking site, gB receptor plays a crucial role in surfing [14]. When bound to a cell, the virus has the ability to increase the number of filopodia by activating RhoA GTPase, Cdc42, in an attempt to approach the cell body [15]. After the virus reaches the cell body and approaches the desired receptors, it can enter the cell via fusion or endocytosis. 

### 2.2. Fusion

There are two key phases in the process of fusion. During Phase I, the viral and the host membranes are brought into close apposition. In phase II the fusion pore forms and expands, resulting in completion of the fusion process [16,17]. The fusion process is initiated by the interaction of gD with one of its receptors which in turn triggers a cascade of events that lead to membrane fusion. Structural studies indicate that a conformational change due to receptor binding in gD signals the pre-complexed gH/gL to undergo conformational change to its post-complexed structure [18]. The first step of this process requires interaction of gD with gH/gL that is followed by gB and gH/gL interaction to form the fusion pore and eventual entry of virus into the cytoplasm (phase II) [19].

#### 2.2.1. Phase I

The interaction of gD with its receptor allows for tight anchoring of virus to the plasma membrane of the host cell, bringing them into close juxtaposition [20]. HSV uses HVEM, nectin-1, nectin-2, or 3‑O-sulfated heparan sulfate as gD receptors for membrane-fusion [21]. The ectodomain of gD contains both receptor and glycoprotein binding sites as soluble forms of gD are sufficient to turn noninfectious gD-null HSV mutant to a wild type-like infection [18]. The N-terminal region of the ectodomain consists of receptor binding sites and the C-terminal region encompasses glycoprotein binding sites. When not bound to one of its receptors, gD has a closed conformation structure in which the C-terminus is anchored near the N-terminus, concealing the receptor binding sites [22]. Upon receptor binding to the N-terminus of gD, the C-terminus is simultaneously released leading to an open conformation [23]. In phase II, the C-terminus activates heterodimer gH/gL into a form that interacts and triggers gB fusogenic activity [24].

#### 2.2.2. Phase II

Glycoproteins H and L form a noncovalent complex on the virion envelope to be functional fusogenic proteins in viral entry [25,26,27]. It may not be essential that the association of gB-gH/gL requires gD binding to its receptor [19,28]. However, gD receptor binding is found to promote the formation of gB-gH/gL complex [19]. Initially, gH has been thought to be involved in forming a hemifusion complex [29], however, recent studies have shown that gH does not resemble any fusogenic proteins and might only assist other glycoproteins in forming a hemifusion complex [30]. It could be involved in the kinetics of fusion and be responsible for a rate-limiting ﬁrst stage in HSV-1 fusion as specific mutations to the ectodomain with unbroken functional activity have shown to be able to perform fusion, but at a slower rate [31]. The ectodomain is explicitly known to bind to receptors on the cell surface as soluble gH/gL has shown to block infection in a dose dependent manner [32]. However, specific receptors that bind to the heterodimer have not been clearly identified yet. There is evidence that the receptor could be αvβ3 integrin [33], but supplementary studies indicate that it is not necessarily so and that other proteins might also be involved [32].

The gH/gL heterodimer directly binds with gB to complete the fusion process. Interestingly, gB and gH/gL are not required to be present on the same membrane as fusion has been described between cells when these glycoproteins are not present on the same cell surface [34]. gB is the most highly conserved entry glycoprotein in herpesviruses and is required for initial viral attachment and latter viral entry. The glycoprotein gB has first been reported to be involved in membrane fusion when it was observed that mutations in the cytoplasmic domain of gB resulted in a syncytial phenotype [35]. Analysis of crystal structure of gB shows that it has a similar fold and fusion loop to known fusion proteins such as vesicular stomatitis virus (VSV) G protein [36]. Unlike other fusogen proteins, which are distinctly classified as either class I (e.g., influenza virus hemagglutinin) or class II (e.g., flavivirus E protein), gB shares properties of both groups. This places gB in a newly defined group of fusogens called class III.

The fusion loops of gB are first inserted into the host membrane which is then followed by the interaction with gH/gL [16]. The interaction of gH/gL with gB helps convert the prefusion glycoprotein to a postfusion conformation that is capable of forming the fusion pore [34]. This binding possibly occurs prior to fusion, as some anti HSV gB antibodies can block fusion without disrupting this complex [37]. Fusion pore completes the fusion process and the virion particles, along with the tegument, enter the cytoplasm.

### 2.3. Endocytosis

The ability of HSV to enter multiple cell types, using multiple pathways, maximizes its chances of infecting multiple cell types in a host and achieving successful infection. Penetration from intracellular vacuoles has the advantage of leaving no viral glycoproteins exposed on the cell surface for immune detection [38]. There are two ways a virus enters a cell via endocytosis: a pH dependent and a pH independent pathway [39,40,41]. Unlike fusion, both processes are energy dependent and use gD glycoprotein [39]. The endocytic pathway of virus is found to be similar to phagocytosis as the entry of virus involves rearrangement of actin cytoskeleton, trapping of the virions by membrane projections, trafficking of virions in large phagosome-like vesicles, and utilizing RhoA GTPase activation pathway, which are characteristics of phagocytosis [42]. Post-endocytosis, a crucial step after entry of enveloped viruses in both processes is the fusion of viral and cellular membranes leading to release of the nucleocapsid into the cytoplasm. An acidic compartment is not required for this fusion in the pH independent pathway [39].

It still remains a mystery why one pathway is used over the other, given that the same glycoproteins are involved for both entry pathways. A low pH environment cannot trigger the fusion machinery and a gD receptor is always needed [41]. One possibility to undergo endocytosis is made by the presence of dense cortical cytoskeleton near the plasma membrane, which prevents entry by direct fusion [38,43]. The cell type could also be another factor. CHO cells, HeLa cells, and keratinocytes are thought to permit HSV-1 entry through a pH-dependent pathway after endocytosis of cell bound viruses [40,41]. On the other hand, viruses directly fuse to the plasma membrane of Vero and neuronal cell lines [40,41,44].

## 3. Therapeutics

The development of novel strategies to eradicate HSV is a global public health priority and yet, despite this fact, there have been very few major breakthroughs in the treatment or prevention of the virus since the introduction of acyclovir in the 1980s. Entry based therapies have significant potential for treating herpes infection. Reduced entry also translates to decreased replication and spread to other cells. N-docosanol is one of the entry preventing drugs that is used for HSV treatment. It is a saturated alcohol that inhibits HSV infection by interfering with entry and its specific mechanism of action has not been fully characterized yet [45]. It is a highly lipophilic compound and is thought to target viruses with lipid-containing envelopes to inhibit fusion [46]. Although very effective, ten percent docosanol cream is the only, FDA-approved, over the counter therapy for herpes labialis, but not for treatment of recurrent genital herpes or ocular infections [45]. Since the latter two diseases are predominantly the cause of global burden and result in detrimental symptoms of virus, more entry-based therapies are required. Mentioned below are entry-preventing drugs that show promise in treating various manifestations of HSV-1 and HSV-2. 

### 3.1. Receptor Targeting Therapeutics

**Figure 1:**
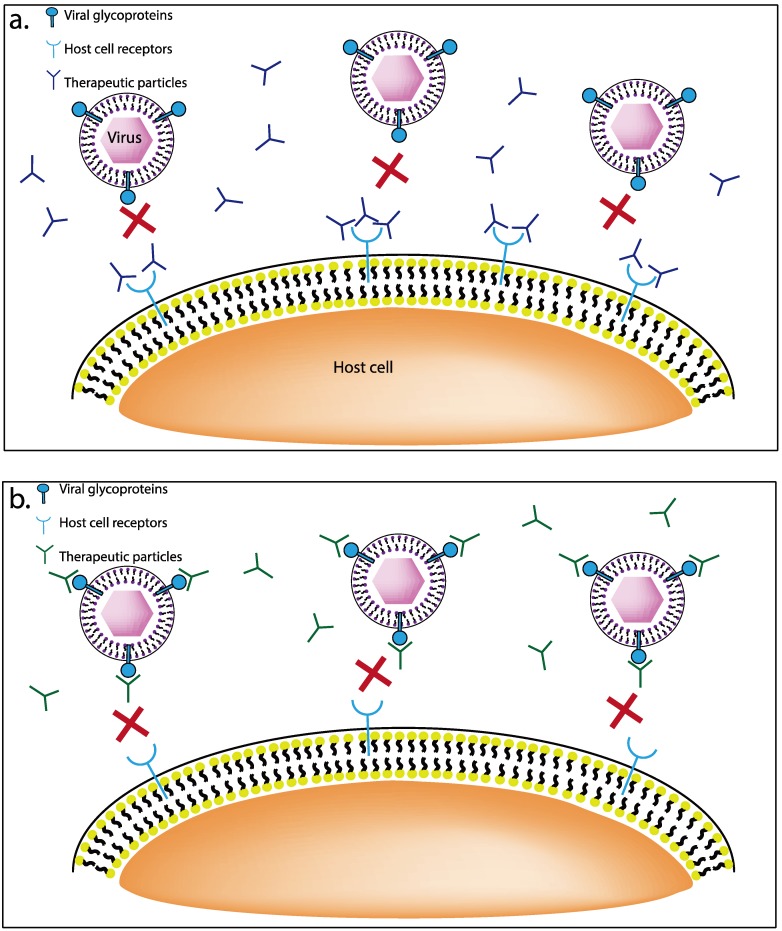
(**a**) Schematic representation of therapeutics that target the receptors on the host cell surface. These therapeutics are mostly cationic or positively charged, and have the ability to bind to negatively charged groups such as sulfates and carboxyl groups of host receptors. (**b**) Schematic representation of therapeutics that target the viral glycoproteins. The anionic charged therapeutics bind to the viral glycoproteins and block the interaction of virus and host.

Several new classes of cationic charged inhibitors have been recently described (Figure 1), and many of them bind HSV entry receptors or glycoproteins present on the host cell surface and demonstrate prophylactic and therapeutic efficacies. A few such agents are discussed below. 

#### 3.1.1. G1 and G2 Anti-Heparan Sulfate Peptides

Two recently isolated peptides, G1 and G2, were found to bind to cell surface receptors HS and 3‑OS-HS. Targeting one of these two receptors has been shown to block HSV-1 infection *in vitro* in CHO K1, HeLa, and corneal fibroblasts cells in a dose dependent manner (9). G2 was also found to absolutely inhibit viral replication and to hinder cell-to-cell spread of HSV-1 by 70%–80% using HSV‑1 (9) and HSV-2 (2).

Prophylactic experiments with the peptides prevented infection in eyes and genitals of mice. Prophylactic eye drops were found to block infection within the mouse cornea and prevented detachment of the corneal epithelium (9). When these peptides were used as pretreatment in mice genitalia prior to HSV-2 infection, the number of lesions caused by the virus on infected mice genitalia was reduced drastically (2). The target of these peptides, HS, is widely expressed and on all types of cells, and is up regulated in HSV-1 and HSV-2 infected cells (2) (9). This upregulation is also accompanied by hyper modifications to 3-OS HS, which increases peptide affinity by 200% (2). Since, the peptides preferentially target infected cells, healthy cells and any associated cytotoxicity may be avoided.

#### 3.1.2. Apolipoprotein E

Apolipoprotein E (apoE) is a 34-kDa glycoprotein that can bind to heparan sulfate proteoglycans (HSPGs) in the extracellular matrix, which is crucial for viral attachment to cells [47]. A tandem repeat dimer peptide, apoEdp, a derivative of apoE, is has been shown to exhibit antiviral activity against HSV-1, HSV-2, and HIV, *in vitro* [48]. In *in vivo* experiments in mice, reduced corneal disease in pretreated apoEdp-treated eyes correlated with downregulated expression of proinflammatory cytokines, IL-1α, IL-1β, IL-6, TNF-α, IFN-γ, and VEGF, within 24 hours after treatment. Topical treatment of 1% apoEdp was potent as both an antiviral, measured through reduction of viral titers, and as anti-inflammatory, measured through the reduction of pro-inflammatory and angiogenic cytokines, without any local or systemic toxicity in mice [48]. The therapeutic effect was similar to topical application of 1% trifluorothymidine (TFT), a drug currently being used ubiquitously against HSV-1 infections. Topical treatment of 1% apoEdp has demonstrated therapeutic efficacy against HSV-1 and HSV-2 induced epithelial keratitis in rabbits. The severity of neovascularization and corneal opacity was significantly reduced when compared with placebo treated rabbit eyes [48].

#### 3.1.3. AC-8

Another potential cationic peptide AC-8, an IgG FAB fragment, shows promise as an HSV antiviral that binds to a gD receptor. A study has shown that AC-8 effectively delayed corneal opacity and decreased tear film viral titers, corneal vascularization, and epithelial keratitis in mice. Given the essential role of gD in HSV viral entry, AC-8 was successfully targeted and prevented infection against several HSV-1 strains of KOS and F, indicating that AC-8 has a broad-spectrum activity across strains [49]. AC-8 is mildly less effective in combating infection, but conveniently surpasses TFT when it comes to cytotoxicity and repeated use. TFT induces mild toxic changes such as intracellular edema of the basal cell layer, mild thinning to the overlaying epithelium, and reduces the strength of stromal wounds [50]. Repeated administration of TFT over 21 days is not recommended. On the other hand, no cellular damage or inflammation was seen after repeated therapeutic use of AC-8 [49]

#### 3.1.4. Aptamers

Aptamers are nucleic acid based molecules that are capable of specifically binding to a target molecule with high affinity [51,52]. They have the ability to fold into a variety of complex, sequence specific tertiary conformations, similar to that of antibodies and hence have the potential to bind to a wide range of targets. Many minimized aptamer sequences are short RNA stem loops. They are therefore a bridge between traditional small molecular weight drugs and macromolecular therapeutics, such as antibodies and their fragments [51,52]. Using Systematic Evolution of Ligands by Exponential Enrichment (SELEX), several successful functioning aptamers have been isolated against viruses. For example, aptamers were isolated against the gp120 envelope glycoprotein of human immunodeficiency virus (HIV) type 1 [53,54], gB and gH glycoproteins of two strains of cytomegalovirus (L13 and L19, respectively) [55], and more recently against gD glycoproteins of HSV-1 [56] and HSV-2 [57]. 

In a study by Gopinath *et al*., an RNA aptamer that inhibited the interaction of gD with its receptors was isolated. Plaque assays in Vero cells confirmed reduced interaction of gD with HVEM and nectin‑1 with an IC_50_ value of lower than 50 nM. In another study, Moore *et al.* were able to isolate an RNA aptamer that inhibited the interaction of gD with its receptors nectin-1 and HVEM. Plaque assays and B-galactosidase experiments with Vero cells showed that the aptamers are effectively able to neutralize HSV-2. The IC_50_ value of the aptamers ranged from 20 to 50 nM. These aptamers were able to neutralize HSV-2 to a similar extent to a gD binding antibody [57]. The other advantage of using aptamers is their high specificity towards target molecules, as aptamers from either of the mentioned experiment did not interact with the gD structure of the other strain of virus. This is in stark contrast with neutralizing antibodies as targeting gD protein generally show cross-reactivity and inhibit both strains of virus HSV-1 and HSV-2 [58].

#### 3.1.5. Dermaseptins

Dermaseptins, a family of eight closely related peptides, are isolated from the skin of a tree dwelling South American frog and possess anti-viral properties. These compounds are polycatioic peptides, composed of 28–34 amino acids long, unstructured, and folded into amphipathic a-helices upon contact with lipid membranes [59,60,61]. Primarily used as an antimicrobial, a specific group S1‑S5, with lysine rich peptides, has shown to provide antiviral activity against HSV-1 [62] and HSV‑2 [63]. They are known to interrupt the initial virus-cell interaction, possibly through ionic interactions between their positively charged amino acids, and the negatively charged sulfate and carboxyl groups in heparan sulfate constitute the major binding interactions [63]. 

Among the five dermaseptins, S4 was found to the most effective against HSV-1 and HSV-2. *In vitro* experiments of S4 with HSV-1 decreased virus yield of acyclovir sensitive and resistant HSV‑1 significantly below cytotoxic levels [62]. In a more recent study, the activity of dermaseptin K4K20S4, which is synthetically modified to increase net positive charge of the peptide exhibited dose‑dependent levels of inhibitory effects against sensitive and resistant HSV-2 strains with lower cytotoxicity levels [63]. Furthermore, the activity of dermaseptins is maintained at low pH conditions [64], suggesting that the peptide could be active in the genital tract. 

Other natural cationic peptides that could potentially be used as HSV antivirals include: defensing, cecropin A, melittin, magainin I and II, and indolicidin [65,66,67,68].

### 3.2. Viral Glycoprotein Targeting Therapeutics

Viral Glycoproteins are necessary for fusion and are largely positively charged. Hence, polyanionic compounds have the capacity to inhibit HSV infection and replication *in vitro* by targeting the high affinity sulfated compound-virus complex [69,70]. Negatively charged polyanions bind to HSV glycoproteins, blocking viral attachment and/or adsorption to cells thereby preventing infection [71]. Described below are some of the most common polyanionic agents with well-examined abilities to control HSV-1 entry and infection. 

#### 3.2.1. Nanoparticles

Targeting the initial interaction between the virus and cellular HS is preferred as numerous viruses use this pathway and this offers the potential of a broad-spectrum antiviral drug. The virus initially binds to the HS with gB and gC to attach to the cell surface as mentioned earlier. Recent advances in nanotechnology have spurred the development of metal oxide nanostructured compounds that have binding affinity to viral glycoproteins. Several nanostructures from metal-based materials have shown antiviral properties such as zinc oxide (ZnO) [72], tin oxide (SnO) [73], liquid filled protein microspheres (PM) [74], and gold nanoparticles capped with mercaptoethance sulfonate (Au-MES) [75]. The cause of this is attraction of negatively charged nanowires, which is similar to the natural target receptors on the cell membrane. It might also be possible to present these compounds as virus trappers that stimulate immune response while providing protection from virus infection as microbicides [73]. The combined effect would lead to improved viral clearance and overall antiviral effectiveness. Reduced entry also translates to decreased replication and spread to other cells. These new drugs that target other critical steps in viral lifecycle, such as entry and cell-to-cell spread, can help reduce the likelihood of viral resistance to therapeutic agents.

#### 3.2.2. K-5 Compounds

HSV-2 is a major risk factor in the acquisition of HIV-1 infection [76]. Infection with HSV-2 increases the likelihood of becoming HIV-1 positive by two to threefold, irrespective of clinical symptoms [76]. Therefore, therapeutics aimed at preventing the transmission of HSV-2 and HIV simultaneously could prove promising. Among many polyanionic compounds, K-5 compound therapeutics effectively addresses this issue. Previously characterized as potent, broad-spectrum anti‑HIV-1 compounds [77], K5 derived compounds have inhibited both HSV-1 and HSV-2 infection by free virions, as well as cell-to cell virus spread *in vitro* [78]. The mechanism of action of K5 compounds seem to form complexes and interact with gB, and thus inhibiting initial virus binding and subsequent fusion as well as cell-to-cell spread of infection [69]. The particular compounds of interest, K5 polysaccharides of *E. coli* have the same structure as biosynthetic precursor of hepatin/heparan sulfate, N-acetyl heparosan, with slight variations [78]. The two compounds, K5-N,OS(H) and its epimerized product Epi-K5-OS(H), blocked HSV-2 infection at both entry and post-entry levels. At the entry level, the compounds bound more effectively to gB than to gC and limited cell-to-cell spread of the HSV-2 [79]. The molecular basis to the inhibition at post entry levels is currently unknown but likely impedes the transport of virus across the plasma membrane. These anti-HSV along with anti‑HIV activities, make these compounds suitable for development as antiviral therapeutics for preventing sexual transmission of both HSV and HIV.

#### 3.2.3. Compound SP-510-50

Compound SP-510-50, which is an antibody to glycoprotein gD and has shown to provide antiviral efficacy against HSV-1 infection. *In vitro* experiments in Vero cells show that the drug prevents infection in a dose dependent manner. *In vivo* experiments in rabbits treated for ocular epithelial HSV keratitis, showed comparable antiviral efficacy at a lower dose (four times a day) when compared to the existing drug of choice trifluridine (TFT) (nine times a day). Similar to the *in vitro* studies, this treatment reduced corneal surface viral tiers and disease symptoms in a dose-dependent manner [77,80]. Other potential therapeutic polyanionic compounds are PRO 2000, polystyrene sulfonate, polymethylenehydroquinone sulfonate, and cellulose sulfate [69].

#### 3.2.4. Dendrimers

Dendrimers are macromolecular compounds that comprise a series of branches around an inner core that can comprise of carbohydrate or amino acid structures. Dendrimers are attractive anti-viral therapeutics because of their size (nanomolar), their ease of preparation and functionalization, their ability to display multiple copies of surface groups, and their ability to target either viral glycoproteins or receptors [81,82]. Their surface functional groups enable multiple drug molecules to be attached to the surface, enabling a high drug payload, compared to typical linear polymers [83]. There are generally two types of dendrimers based on the makeup of the core, namely glycodendrimer and peptide-dendrimer. Dendrimers have successfully been used as carriers for drug delivery against HSV‑1 and HSV-2. 

One of the most successful therapeutic dendrimers is SPL7013 and has demonstrated efficacy against herpes simplex virus in *in vitro*, and in animal models. SPL7013 contains specifically designed polyanionic surface that allows it to target virus glycoproteins. Gong *et al.* [84] evaluated SPL7013 for its efficacy against herpes and for cell cytotoxicity. Incubating Vero cells with SPL7013 prior to the addition of viral inoculum showed reduction in plaque assays with the EC_50_ values of 2.0 µg/mL for HSV-1 and 0.5 µg/mL for HSV-2. SPL7013 was not toxic up to highest concentrations tested 10,000 µg/mL in Vero cells. Effects on cell proliferation were tested on two epithelial cell lines (Hela‑229 and Hep-2) in both stationary and dividing phases, and found the 50% cytotoxic concentrataion (CC50) to be greater than 10,000 µg/mL. The effect of SPL7013 drug concentration and prophylaxis was evaluated in mice with HSV-2 genital infections [85]. A dose related response was showed with mice being protected from infection at various concentrations. 

Multiple phase I human trials have been conducted to evaluate safety, tolerability, and systemic pharmacokinetics of SPL7013 Gel (Vivagel®) in healthy adults. None of the women [86,87] or men [88] tested at the highest dose had detectable plasma levels of SPL7013, indicating that it was not absorbed systemically. Colonoscopic assessment found no clinically significant findings of vulval, vaginal, or cervical inflammation, or other pathology determined to be related to the study gels. The levels of potential symptoms of genital irritation reported during the study were low and could be attributed to the gel contents rather than the active ingredient SPL7013. 

Another glycoprotein-targeting dendrimer utilizes a peptide gH625 as branches and has shown to have therapeutic effect against HSV-1 and HSV-2 [89]. This peptide is derived from viral fusion protein gH and has been shown to exhibit antiviral properties. The peptide is thought to bind to the one or all of the essential fusion proteins, especially gH, and inhibit the viral fusion process. Experiments revealed antiviral activity through a viral yield assay on both HSV-1 and HSV-2 and showed a consistent decrease in replication efficiency for both viruses. The IC_50_ value of the peptidodendrimer was 100 and 300 nM against HSV-1 and HSV-2, respectively.

Polycationic dendrimers, SB105 and SB105_A10 are promising candidates for the development of novel topical microbicides for the prevention of HSV infections. Luganini *et al.* [90] report on these dendrimers to potently inhibit the *in vitro* replication of HSV-1 and HSV-2 by a mechanism that involves the inhibition of virion attachment to cell-surface heparan sulfate proteoglycans (HSPGs). Pretreatment of Vero cells with SB 105 and SB105_A10 peptides one hour before infection produced a significant concentration dependent inhibition of both HSV-1 and HSV-2. The IC_50_ values of both dendrimers were 0.4 for HSV-1. The IC_50_ values for HSV-2 were 1 and 0.8 µM for dendrimer SB105 and SB105_A10, respectively. Cytotoxicity of the dendrimers was not observed in target cells at maximum concentration tested at 100 uM. Acidic treatments of pH 3.0 and 4.0 did not affect the stability of either SB105 or SB105_A10 or their ability to inhibit infection. This elucidates their functional role in the physiological environment of the genital tract.

Furthermore, marine organisms, plants, and other bacteria are potential sources of entry inhibiting antiviral compounds against herpes simplex virus. A review by Vo *et al.* [91] has summarized promising anti-HSV agents found in a algae, sponges, tunicates, echinoderms, mollusks, shrimp, and fungus, some of which are entry preventing therapeutics. Aqueous neem bark extract (NBE) from neem plant *Azardirachta indica*, acts as a potent inhibitor during initial viral entry and later glycoprotein mediated cell-to-cell fusion [92]. Cyanovirin-N (CV-N), a sugar binding antiviral protein isolated from cyanobacterium *Nostoc ellipsosporum,* also has to shown to inhibit viral fusion, reduced cell-to-cell fusion and polykaryocytes formation [93].

### 3.3. Targeting Downstream Signaling Mechanism

Another phase of infection that could be targeted by HSV therapeutics is the downstream signaling mechanism during entry. Many viruses manipulate the cytoskeleton of the host during crucial aspects of their lifecycle [94]. Live cell imaging has demonstrated that retroviruses such as MLV, avian leucosis virus, and vesicular stomatitis virus associate with dense microvilli and/or filopodia of polarized epithelia and move on to the cell surface, known as surfing, in an actin and myosin‑dependent manner prior to internalization [95,96]. Actin cytoskeleton is used by HSV-1 during phagocytic uptake by primary cultures of human corneal fibroblasts and surfing in retinal pigment epithelial cells [42]. PI3K are a family of heterodimeric enzymes, which activate lipids as second messengers that regulate the phosphorylation of various kinases such as Akt/PKB, cyclic AMP-dependent protein kinase A, a few protein kinase C isoforms, and ribosomal S6 kinases p70 and p85 [97]. The herpes virus exploits PI3K enzymes during filopodia induction and cell-to-cell fusion.

Filopodia induction facilitates viral entry by increasing the cell surface area for surfing and targeting cells [14]. A PI3K inhibitor successfully inhibited HSV-1 viral entry in a dose dependent manner in RPE, HeLa, and CF cells. It prevented viral entry in various herpesviruses such as HSV-1 (alpha-herpesvirus), cytomegalovirus, and human herpes virus-8, suggesting that the effect of PI3K signaling may be universal among herpesviruses. The inhibitor works by targeting filopodia formation and glycoprotein-mediated cell-to-cell fusion [98]. 

## 4. Conclusion

Humans harbor HSV in their neurons and non-neuronal sites. Most humans exhibit asymptomatic shedding based on studies from tears, saliva, and vaginal secretions, and accounts for the high percentage of humans infected. Current therapeutics against HSV predominantly include nucleoside analogs such as triflurothymidine (TFT), and topical/oral acyclovir. Approximately, US $17.7 million is expended annually to treat 59,000 new, and 29,000 recurrent, cases of herpetic eye diseases in the United States. Oral acyclovir alone costs $8,532 US dollars per ocular episode averted. If antiviral prophylaxis were more effective, the cost per infection would decrease by 51% [99]. Therefore, aiming for prophylactic measures in patients would be more cost effective than prescribing acyclovir. To conclude, this paper discusses and provides information of known entry mechanisms and potential new therapeutics that target the entry stage of infection. A better understanding of viral entry mechanisms in the last decade has facilitated emergence of a large number of new lead candidates against the virus. While several of the candidates discussed in this paper show strong promise for development as effective prophylactic agents, certainly more *in vivo* studies, including human trials, are warranted to bring them to clinical applications. HSV may not be a curable virus but the new therapies will certainly make it much easier to manage. 
